# Pancreatic Cyst Size Measurement on Magnetic Resonance Imaging Compared to Pathology

**DOI:** 10.3390/cancers16010206

**Published:** 2024-01-01

**Authors:** Daniel Jeong, Brian Morse, Stuart Lane Polk, Dung-Tsa Chen, Jiannong Li, Pamela Hodul, Barbara A. Centeno, James Costello, Kun Jiang, Sebastian Machado, Issam El Naqa, Paola T. Farah, Tri Huynh, Natarajan Raghunand, Shaffer Mok, Aamir Dam, Mokenge Malafa, Aliya Qayyum, Jason B. Fleming, Jennifer B. Permuth

**Affiliations:** 1 Department of Diagnostic Imaging and Interventional Radiology, H. Lee Moffitt Cancer Center & Research Institute, 12902 USF Magnolia Drive, Tampa, FL 33612, USA; brian.morse@moffitt.org (B.M.); james.costello@moffitt.org (J.C.); aliya.qayyum@moffitt.org (A.Q.); 2 Department of Cancer Epidemiology, H. Lee Moffitt Cancer Center & Research Institute, 3011 Holly Drive, Tampa, FL 33612, USA; jenny.permuth@moffitt.org; 3 College of Medicine, University of South Florida, 12902 USF Magnolia Drive, Tampa, FL 33612, USA; stuart.l.polk@vumc.org; 4 Department of Biostatistics and Bioinformatics, Moffitt Cancer Center, 12902 USF Magnolia Drive, Tampa, FL 33612, USA; dung-tsa.chen@moffitt.org (D.-T.C.); jiannong.li@moffitt.org (J.L.); 5 Department of Gastrointestinal Oncology, H. Lee Moffitt Cancer Center & Research Institute, 12902 USF Magnolia Drive, Tampa, FL 33612, USA; pamela.hodul@moffitt.org (P.H.); shafer.mok@moffitt.org (S.M.); aamir.dam@moffitt.org (A.D.); mokenge.malafa@moffitt.org (M.M.); jason.fleming@moffitt.org (J.B.F.); 6 Department of Anatomic Pathology, H. Lee Moffitt Cancer Center & Research Institute, 12902 USF Magnolia Drive, Tampa, FL 33612, USA; barbara.centeno@moffitt.org (B.A.C.); kun.jiang@moffitt.org (K.J.); 7 Department of Clinical Science, H. Lee Moffitt Cancer Center & Research Institute, 12902 USF Magnolia Drive, Tampa, FL 33612, USA; sebastianmd98@gmail.com (S.M.); ultiangel123@aim.com (P.T.F.); 8 Department of Machine Learning, H. Lee Moffitt Cancer Center & Research Institute, 12902 USF Magnolia Drive, Tampa, FL 33612, USA; issam.elnaqa@moffitt.org; 9 College of Medicine, University of Florida, 1600 SW Archer Rd, Gainesville, FL 32610, USA; trihuynh331@gmail.com; 10 Department of Cancer Physiology, H. Lee Moffitt Cancer Center & Research Institute, 12902 USF Magnolia Drive, Tampa, FL 33612, USA; natarajan.raghunand@moffitt.org

**Keywords:** magnetic resonance imaging, intraductal papillary mucinous neoplasm, size measurement

## Abstract

**Simple Summary:**

Intraductal papillary mucinous neoplasms are non-invasive precursor lesions for pancreatic cancer that are often surgically resected when certain diagnostic criteria are met. While multiple cyst features play a role in determining their resectability, cyst size is an important factor. The aim of this study is to compare the cyst size on seven commonly-obtained MRI sequences with surgically-resected specimen pathology size in a retrospective cohort of 52 patients. The maximum MRI size across the sequences correlated well but tended to overestimate length compared to pathology measurement.

**Abstract:**

BACKGROUND: While multiple cyst features are evaluated for stratifying pancreatic intraductal papillary mucinous neoplasms (IPMN), cyst size is an important factor that can influence treatment strategies. When magnetic resonance imaging (MRI) is used to evaluate IPMNs, no universally accepted sequence provides optimal size measurements. T2-weighted coronal/axial have been suggested as primary measurement sequences; however, it remains unknown how well these and maximum all-sequence diameter measurements correlate with pathology size. This study aims to compare agreement and bias between IPMN long-axis measurements on seven commonly obtained MRI sequences with pathologic size measurements. METHODS: This retrospective cohort included surgically resected IPMN cases with preoperative MRI exams. Long-axis diameter tumor measurements and the presence of worrisome features and/orhigh-risk stigmata were noted on all seven MRI sequences. MRI size and pathology agreement and MRI inter-observer agreement involved concordance correlation coefficient (CCC) and intraclass correlation coefficient (ICC), respectively. The presence of worrisome features and high-risk stigmata were compared to the tumor grade using kappa analysis. The Bland-Altman analysis assessed the systematic bias between MRI-size and pathology. RESULTS: In 52 patients (age 68 ± 13 years, 22 males), MRI sequences produced mean long-axis tumor measurements from 2.45–2.65 cm. The maximum MRI lesion size had a strong agreement with pathology (CCC = 0.82 (95% CI: 0.71–0.89)). The maximum IPMN size was typically observed on the axial T1 arterial post-contrast and MRCP coronal series and overestimated size versus pathology with bias +0.34 cm. The radiologist interobserver agreement reached ICCs 0.74 to 0.91 on the MRI sequences. CONCLUSION: The maximum MRI IPMN size strongly correlated with but tended to overestimate the length compared to the pathology, potentially related to formalin tissue shrinkage during tissue processing.

## 1. Introduction

Intraductal papillary mucinous neoplasms (IPMNs) are non-invasive, cystic neoplasms of the pancreatic main duct and/or its side branches. A significant proportion have the potential to harbor high-grade disease and undergo malignant transformation to invasive pancreatic ductal adenocarcinoma [1,2,3,4]. Although IPMNs can be asymptomatic, they are increasingly being incidentally detected on imaging, with a prevalence of 19.9% on magnetic resonance imaging (MRI) and 2.6% on computed tomography (CT) [5,6]. With such a high frequency of incidental detection of IPMNs on imaging, there has been controversy within the radiology community on how to accurately characterize them as benign or malignant. Currently, most institutions use the International Consensus Guidelines (ICG) (also called the Fukuoka Consensus Guidelines, or the Fukuoka Criteria) to provide the basis for risk stratification of IPMNs on imaging [7,8]. ICG consists of categories called ‘worrisome features’ and ‘high-risk stigmata’ (WF/HRS), which include radiologic and clinical features used to assess the malignant potential of IPMNs. Contingent upon the presence or absence of certain ICG criteria, a radiologist may classify an IPMN as benign or malignant. Patients with a benign IPMN will be assigned for surveillance with imaging follow-up, whereas a patient with a malignant IPMN is recommended for further work up and/or surgical resection.

In addition to the assessment of high-risk stigmata such as the presence of main duct involvement and/or mural nodules, size measurement is an important criterion in stratifying pancreatic IPMN cases into surgical resection or imaging follow-up groups. It has been shown that IPMNs with a size measuring >3 cm on imaging are at a much greater risk of harboring high-grade or malignant pathology compared to those <3 cm [9,10,11,12]. Therefore, an IPMN size measuring >3 cm is categorized as a ‘worrisome feature’ under the ICG and indicates further workup with endoscopic ultrasound (EUS) and/or surgical resection [7]. Given that patients with low-grade IPMNs have an excellent 5-year survival (94–100%) compared to those with high-grade/invasive IPMNs (31–65%), it is imperative to make an accurate and prompt pathologic determination [10,13,14,15,16]. Furthermore, there is a risk that comes with the surgical resection of IPMNs (post-operative morbidity of 40% and mortality of 2.3%) and therefore surgery should ideally be reserved for those with the highest risk for having high-grade IPMN pathology [17]. 

There is significant variability in measuring IPMN size both between and within different imaging modalities [18,19]. However, MRI and CT IPMN size measurements have shown moderate agreement with post-operative pathology and exhibit similar inter-observer variability when measuring IPMN size (ICC = 0.81–0.86 for MRI, ICC = 0.85 for CT) [18,19,20,21]. While MRI and CT have both proven to be efficacious in accurately predicting IPMN malignancy (MRI AUC = 0.82–0.86, CT AUC = 0.82–0.83), MRI exhibits certain advantages over CT when evaluating IPMNs [22,23,24]. MRI performs better than CT in classifying IPMN type and identifying the extent of the disease, regarding ductal connections, main duct involvement, and branch duct cysts [25]. MRI also allows the accurate identification of associated solid nodules ≥5 mm which correlate with lesion malignancy [19]. Additionally, it has been shown that CT is unable to visualize 22% of the IPMNs visualized on MRI, as well as 3% of IPMNs greater than 15 mm visualized on MRI [26]. 

Although consensus society statements provide guidance when MRI is used to evaluate pancreatic IPMNs, there is no single universally-accepted imaging sequence which provides optimal size measurements [27,28]. Previous authors support reporting longest axis cyst size on T2W coronal or axial imaging or magnetic resonance cholangiopancreatography (MRCP) in an attempt to report a highly reproducible and maximal diameter [21,27]. Standard MRI exams can have over 20 series in which size measurements of a single lesion can vary considerably across sequences. The MRI sequence chosen to measure the maximum IPMN long-axis diameter is important and can sometimes stray from the guidelines, potentially explaining the moderate values reported for inter-observer agreement in measuring IPMN size on MRI (kappa = 0.59–0.70) [21,29]. However, employing IPMN measurement standards has been shown to improve the agreement within a group of radiologists [21,27]. These include: measuring from outer-wall to outer-wall on coronal T2 weighted images, using axial images for localization, using maximal intensity projection (MIP) MRCP images when IPMNs are poorly visualized on T2 weighted imaging, and excluding a neck or linear ductal connection from measurement [21].

Previous studies show a discrepancy between radiological and pathologic size measurements of hepatic and renal tumors and, likewise, studies involving pancreatic tumors have illustrated a similar lack of correlation between radiological measurement and pathologic size [18,30,31,32,33]. For example, Maimone et al. used MRCP to measure IPMN long-axis diameter in comparison to pathology and reported size differences ranging from 2–44 mm with a mean difference of 5.88 mm, while Lee, Y.S. et al. used a T2 weighted MRI sequence to conduct a similar study which found size differences ranging from 2–24 mm with a mean difference of 8.65 mm [18,31]. Evidently, there is a need for verification that recommended sequences for IPMN size measurements correlate with pathologic size. Increased accuracy in IPMN size measurement can improve the risk stratification of IPMNs using the ICG and therefore lead to more appropriate clinical management. The purpose of this study is to compare the agreement between IPMN long-axis diameter measurements including maximum size on seven commonly-obtained MRI abdomen sequences and pathologic gross specimen size measurement. 

## 2. Materials and Methods

Patients with surgically resected IPMNs who were treated at Moffitt Cancer Center (Tampa, FL, USA) between 2007 and 2018 were included in this IRB-approved HIPAA-compliant retrospective cohort with a waiver of consent. Ethics committee approval was not required for this study. Surgical risk stratification was generally based on ICG risk stratification criteria. Fifty-two subjects with pathologically proven IPMN and available preoperative MRI were evaluated. For patients with a multifocal disease, the largest cyst was evaluated. On MRI exams, long-axis diameter tumor measurements of the most concerning pancreatic lesion were performed, extending from outer wall to outer wall on each of the following seven sequences: T2w Axial, T2w Fat-Saturated (FS) Axial, T2w Coronal, MRCP Coronal, T1w Arterial Axial, T1w Venous Axial, Diffusion Weighted Imaging (DWI) low B Axial. 

### 2.1. MRI Techniques 

**Figure 1:**
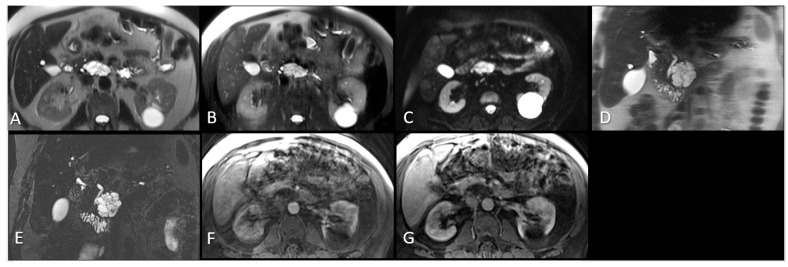
MRI sequences evaluated in this study. (**A**). Axial T2W HASTE. (**B**). Axial T2W fat saturated. (**C**). Axial DWI (low b = 50). (**D**). Coronal T2W HASTE. (**E**). Coronal MRCP. (**F**). Axial T1W fat saturated post contrast arterial phase, (**G**). Axial T1W fat saturated post contrast venous phase. The maximum diameter was measured on the coronal MRCP series for this uncinate process pathology proven pancreatic IPMN. MRI: Magnetic resonance imaging. W: Weighted. HASTE: Half-Fourier single-shot turbo spin-echo. DWI: Diffusion weighted imaging. IPMN: Intraductal papillary mucinous neoplasm. MRCP: Magnetic resonance cholangiopancreatography.

All MR examinations were performed on 1.5 T MRI scanners: n = 36 (Siemens Medical Solutions, Erlangen, Germany); n = 7 (Philips Medical Systems, Amsterdam, The Netherlands); n = 5 (General Electric Medical Systems, Waukesha, WI, USA); n = 2 (Hitachi Medical Corporation, Tokyo, Japan); n = 1 (Toshiba-MEC, Tokyo, Japan); n = 1 (Marconi Medical Systems, Cleveland, OH, USA) using standard pulse sequences. T2 weighted axial images: echo time (TE) = 60–184 ms, repetition time (TR) = 437–22,000 ms, flip angle (FA) = 90–180 ms, slice thickness =3.8–10 mm, field of view (FOV) = [310–480] × [233–400] mm^2^, matrix = [192–256] × 512, number of excitations/averages (NEX) = 1–4, in-plane pixel resolution 0.61–1.76 mm. T2 weighted fat saturated axial images TE = 61–125 ms, TR = 710–15,000 ms, flip angle = 68–180 ms, slice thickness = 3.8–10 mm, FOV = [194–310] × [450–480] mm^2^, matrix = [192–256] × 512, NEX (averages) = 1–3, in-plane pixel resolution 0.61–1.76 mm. T2 weighted coronal images: TE = 67–447 ms, TR = 399–1915 ms, flip angle = 90–180 ms, slice thickness = 4–10 mm, FOV = [298–300] × 480 mm^2^, matrix = [224–240] × 512, NEX (averages) = 1–2, in-plane pixel resolution 0.59–1.56 mm. MRCP coronal images: TE = 120–675 ms, TR = 1000–8062 ms, flip angle = 90–180 ms, slice thickness = 1–3 mm, FOV = 194 × [430–450] mm^2^, matrix = 192 × [768–986], NEX (averages) = 1–2, in-plane pixel resolution 0.38–1.77 mm. T1 weighted fat saturated axial arterial phase: TE = 1–16 ms, TR = 3–475 ms, flip angle = 10–90 ms, slice thickness = 2.5–10 mm, FOV = [200–280] × [410–480] mm^2^, matrix = [160–240] × 512, NEX (averages) = 1, in-plane pixel resolution 0.55–1.60 mm. DWI axial images b = 50:TE = 69–71 ms, TR = 3550 ms, flip angle = 90 ms, slice thickness = 8 mm, FOV = 380 × 450 mm^2^, matrix = 256 × 256, NEX (averages) = 1, in-plane pixel resolution 1.48–1.76 mm. The MRI sequences included in this study are shown in Figure 1.

### 2.2. Image Measurement Techniques

MR images were viewed using the GE Universal Viewer Picture Archiving and Communication System (PACS) version 6.0 (GE Healthcare, Waukesha WI, USA). Images from each MRI sequence displaying the maximal pancreatic cystic lesion dimension were viewed at full screen on radiologic diagnostic monitors and the largest diameter was recorded to the millimeter level for each lesion. Two experienced oncologic abdominal radiologists (DJ and BM) performed the measurements. Within each sequence, the exact MRI slice and measurement direction was decided by each radiologist using standard clinical radiological technique, measuring lesions from outer-wall to outer-wall as suggested by previous authors [21]. Radiologists were blinded to pathologic cyst size and consensus MRI measurements. Each MRI sequence of all exams was also evaluated for the presence of one or more WF/HRS by two observers [7]. If one or more WF/HRS was observed on a particular series, this was noted as positive. 

### 2.3. Pathology Data Collection

Long-axis diameter pancreatic cyst measurements were collected during the gross pathology tissue evaluation process following formalin fixation. The lesion location within the pancreas, tumor grade, and type of surgical treatment were also recorded. IPMNs with a histologic grade of low- or moderate-grade dysplasia were considered “low grade” and tumors with high-grade dysplasia or invasive disease were considered “high grade”. 

### 2.4. Statistical Analysis

The agreement of size measurements between MRI and pathology was evaluated using the concordance correlation coefficient. Bias between MRI sequences and pathology was measured using Bland-Altman analysis. Pearson correlation was performed for cyst size on MRI and pathology for this parametrically distributed data, similar to prior methods [19]. The interobserver agreement for MRI tumor measurements was measured between two experienced abdominal oncology specialized radiologists (DJ and BM) using intraclass correlation. 

Each MRI sequence was also evaluated in its agreement with the tumor grade. Kappa analysis was performed to evaluate the agreement between the presence of positive WF/HRS and high-grade pathology. Kappa was also used to evaluate the interobserver agreement for the presence of one or more WF/HRS on each MR sequence. 

## 3. Results

**Figure 2:**
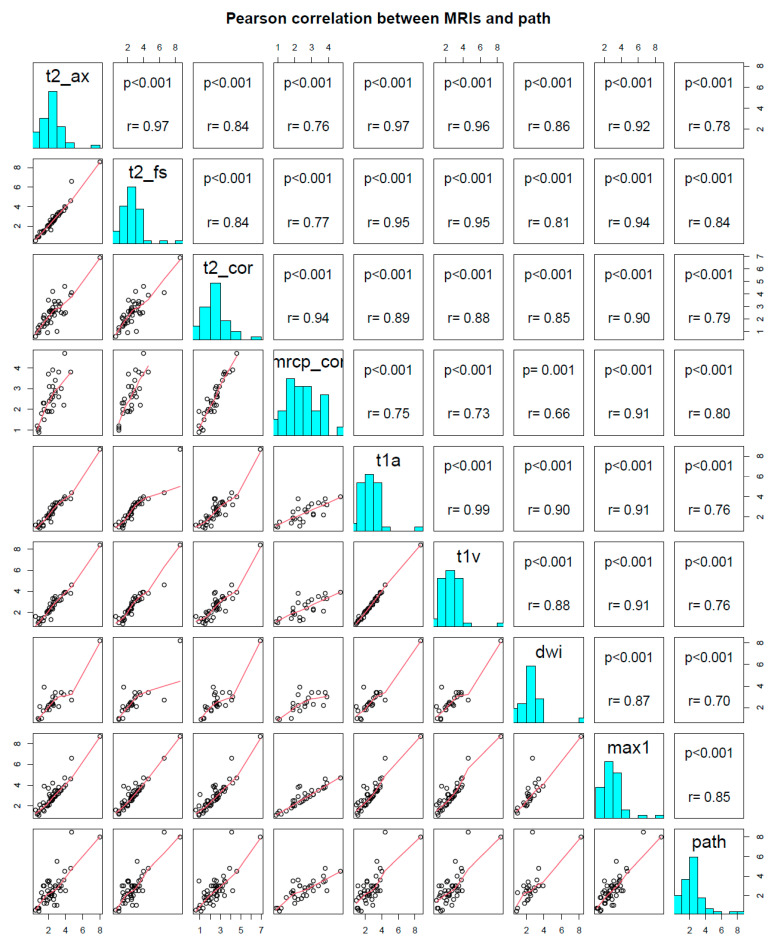
Correlation between MRI and pathology size. The lower left scatterplots show individual size measurements in cm and their correlation with other MRI sequences. The sequence bar graphs (teal) show the IPMN size distribution of lesions within each sequence. The upper right boxes show Pearson correlation (r) and significance (*p* value) between combinations of MRI/path.

The clinical and pathological characteristics of the 52 patients included in this study are summarized in Table 1. The MRI sequences produced mean long-axis tumor measurements ranging from 2.45–2.65 cm (Table 2). The maximum and minimum pathologic size of tumors in this cohort were 8.5 cm and 0.4 cm respectively. The maximum MRI measurement size had a strong agreement with pathology with a CCC (95%CI) of 0.82 (0.71–0.89). The individual MRI sequence agreement with pathology CCC ranged from 0.66 to 0.83 and among the seven MRI sequences the axial T2 weighted fat saturated sequence (T2FS) had the highest agreement with a CCC (95%CI) of 0.83 (0.72–0.90) (Figure 2). Bland-Altman analysis showed standard axial and coronal MRI sequences underestimated size compared to pathology, while MRCP coronal and the maximum measurement overestimated size compared to pathology. T2w Axial MRI underestimated pathology by 0.13 cm or 5%; T2w Coronal MRI size underestimated pathology by 0.10 cm or 4%; and MRCP coronal slightly overestimated pathology by 0.003cm or 0.1%. Complete Bland-Altman results are reported in (Table 2).

There was a strong agreement between radiologists for tumor length measurement on the MRI sequences with ICC ranging from 0.74 to 0.91. The highest interobserver agreement was noted on the low B value DWI sequence with an ICC (95%CI) of 0.91 (0.81–0.95) while the maximum MRI dimension across all examined sequences had an ICC (95%CI) of 0.76 (0.59–0.86). 

**Figure 3:**
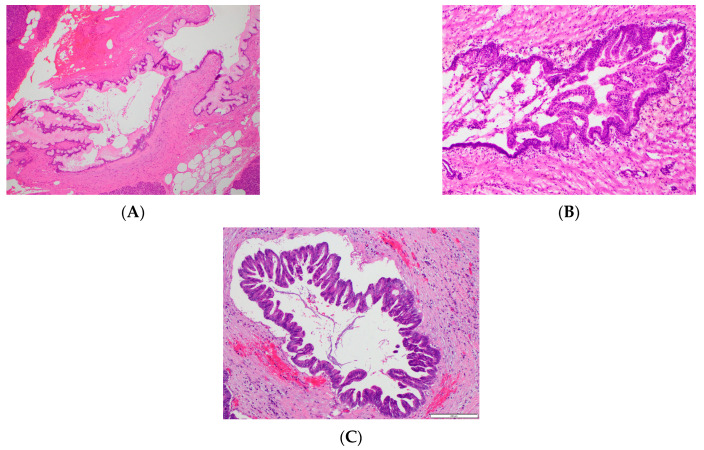
Histopathologic images of IPMNs. H&E-stained samples showing. (**A**). IPMN with low-grade dysplasia, (**B**). IPMN with moderate-grade dysplasia, and (**C**). IPMN with high-grade dysplasia. In this study, low- and moderate-grade dysplasia were considered benign and high-grade dysplasia was considered malignant.

There was moderate agreement between the presence of 1 or more WF/HRS and pathologic high-grade tumor (Table 3). The T2w axial, T2w FS axial, and T2w coronal sequences achieved the highest agreement with pathology, kappa 95%CI of 0.60 (0.36, 0.84). The T1w venous phase axial series had the lower agreement with pathology, kappa 95%CI of 0.45 (0.17, 0.74). There was substantial interobserver agreement in the presence of 1 or more WF/HRS on individual MRI sequences, with T2w sequences having the highest agreement (Table 3). Invasive adenocarcinoma was pathologically noted on 2/52 cases and both cases were correctly assigned as positive for WF/HRS with agreement between reviewers. Of note, both invasive cases were large size > 3.0 cm with significant solid components > 0.5 cm associated with a dominant cystic mass. Histologic examples of IPMN tumor grades commonly encountered in this study are shown in Figure 3. 

## 4. Discussion

In this first study to evaluate MRI size vs. pathology in a retrospective analysis of MRI data from patients who underwent surgical resection for IPMN, we showed that maximum measured MRI IPMN size had strong correlation with pathologic size, but it overestimated pathologic size by 0.34 cm or 11% on average. Because the longest diameter can occur on coronal or axial planes depending on the lesion orientation, the maximum diameter across all sequences was the most clinically applicable data for pathology comparison. Currently, there is no standardized method or sequence for measuring IPMN size on MRI exams. 

Often, radiologists choose the sequence and imaging plane felt to best represent the lesion’s maximal diameter. However, the correlation and bias of maximal MRI size in addition to individual MRI sequence size with IPMN pathology size has not previously been reported. In our study, the MRI average measurements varied 8% across the seven tested sequences. Consequently, for borderline IPMNs these differences can result in measurements above or below the ICG 3 cm “worrisome feature” cutoff based on the sequence used. The maximum IPMN lesion diameter most commonly occurred on the axial T1 weighted arterial phase, with the MRCP coronal accounting for the second most common sequence (Table 2). A previous study from our group showed that MRI tended to over-estimate pathologic cyst size by 0.27 cm or 9.5% [19]. Our results show maximum MRI dimension overestimates pathology size, concordant with previous work. Lesion spatial orientation will affect which imaging plane best represents the true maximum dimension. Although high interobserver agreement is obtained on multiple T2 weighted and T1 post contrast sequences, the maximum lesion diameter across all observed sequences would be most important to report. 

There may be conceptual explanations for why certain MRI sequences show better inter-observer agreement for IPMN size measurement. Our study showed highest inter-observer agreement with the low B-value DWI sequence which tended to have a greater slice thickness than other series and, consequently, fewer slices to choose from during the measurement process. Additionally, the low B-value DWI series offers relatively higher target cystic IPMN signal to background pancreas signal which may have led to more reproducible measurements. However, DWI series offer a limited role in recommended cyst size measurement workflows but they may offer valuable information regarding the presence of restricted diffusion. Conversely, the MRCP coronal series had the lowest inter-observer agreement. Despite also having high target IPMN signal to background pancreas, the MRCP sequence had the smallest average slice thickness of included series which yields the radiologist the most potential room for disagreement on optimal measurement slice selection. 

The T2 weighted sequences provided size measurements with good agreement and acceptable bias when compared to pathology. Additionally, T2 weighted sequences provided the highest agreement with pathology tumor grade along with good interobserver variability measurements. Our study supports previous authors’ suggestions that T2 weighted imaging should play a primary role in IPMN size measurements and tumor grade prediction [21,27].

An important limitation of this study is the phenomenon of tissue shrinkage which occurs with formalin fixation for pathologic evaluation. Given the retrospective nature of this study, more optimal measurements prior to formalin fixation were not available. Studies have demonstrated that tumors of the lung, breast, kidney, head, and neck have exhibited size shrinkage of 4–5% from fresh to formalin-fixed specimens [34,35,36,37]. Renal tumors showed additional shrinkage of 7% from formalin-fixed to histological measurements [37]. Additional studies have shown that certain tumors, such as oral squamous cell carcinomas, have undergone a size shrinkage of 10–11% from fresh to formalin-fixed specimens [19,38]. It is possible that IPMNs are also susceptible to size shrinkage to a similar degree upon formalin fixation before their pathologic size is determined. Therefore, there is potential for measuring bias to occur due to the difference in sizes between in-situ and formalin-fixed specimens. Ideally, an immediate size measurement of fresh specimens prior to formalin fixation would provide the most accurate size comparisons to in-situ measurements by MRI. However, the shrinkage of fresh tumors can be accounted for by applying correction factors of 1.05 for formalin fixed tumors or 1.08 for tumors undergoing histological analysis [35,37]. The fixation duration also influences tissue size shrinkage. Short fixation times (<24 h) have an effect only at the tissue periphery, whereas prolonged fixation (>24 h) has a more pronounced effect on the entire tissue [39,40]. Studies have shown that the typical duration of formalin fixation can range from 6–24 h to 24–48 h, with a minority of instances even greater than 48 h [35,37,41]. However, there is a high internal reliability for measuring pathological tumor size within the same institution due to consistency in tumor shrinkage within tissue-processing laboratories [42]. Additionally, a pancreatic specimen often requires sectioning to find and measure the cyst, which can lead to the collapse of the lesion and possible sub-maximal pathologic size measurement. Tissue-processing-related shrinkage could contribute to relatively higher radiological size measurements compared to pathology; however, a radiological overestimation of size may be somewhat clinically acceptable. 

Another important distinction to note between pathologic and radiological measurements is the structural condition of IPMNs at the time of measurement. Imaging studies measure IPMNs with an intact cyst wall in 3 dimensions, whereas pathology measures IPMNs as an opened cyst in 2 dimensions which may underestimate the actual size of the intact cyst as measured in-situ. Likewise, fine needle aspiration occurring prior to MRI has the potential to decrease cyst size on imaging. In our study, MRIs were obtained prior to any EUS-guided fine needle aspiration. The time passage between preoperative MRI and surgery is another concern. The median time between MRI and surgical resection was 72 days. IPMNs have been reported to have a growth rate between 0.08–1.86 cm per year [43,44]. Additional limitations in this study include its retrospective study design and a small sample size. We recognize that the study is vulnerable to selection bias as the surgically-resected specimens included in this study were those with a preceding pathologic diagnosis. Furthermore, different institutions have varying MRI scanner brands, sequences, and settings, which have been shown to cause variability in tumor size measurement [45]. Larger slice thickness and spacing of each sequence could also introduce variability where the true cyst longest diameter may occur in between slices.

## 5. Conclusions

Our study demonstrated that IPMN maximum MRI size measurements had a strong correlation with pathology size with a strong interobserver agreement; however, maximum MRI size tended to overestimate gross pathologic size. Among the seven MRI sequences evaluated, the maximum IPMN measurement occurred most commonly on the axial T1 arterial post contrast and MRCP coronal series within our cohort. Future larger multi-institutional prospective trials could further define the agreement of MRI measurements including the longest 3D planar diameter and the incorporation with EUS measurements with pre-formalin pathology and determinate applicability in clinical treatment strategies. 

## Figures and Tables

**Table 1:** Clinical and pathological characteristics of IPMN cases in the cohort (*n* = 52).

Variable	
**Mean age, years (SD)**	68.3 (12.8)
**Sex, *n* (%)**	
** Male**	22 (42.3)
** Female**	30 (57.7)
**Location in Pancreas *n* (%)**	
** Head/Neck/Uncinate**	29 (55.8)
** Body/Tail**	23 (44.2)
**Branch Communication *n* (%)**	
** Main**	7 (13.5)
** Side**	23 (44.2)
** Mixed**	22 (42.3)
**IPMN Grade, *n* (%)**	
** Low**	4 (7.7)
** Moderate**	14 (26.9)
** High**	32 (61.5)
** Invasive**	2 (3.8)
**Number of pancreatic cysts**	
** Single**	37 (71.2)
** Multiple**	15 (28.8)

**Table 2:** IPMN tumor size measurements across MRI sequences and pathology (*n* = 52).

	T2w Axial	T2w FS Axial	T2w Coronal	MRCP Coronal	T1w Arterial Axial	T1w Venous Axial	Diffusion Weighted Imaging (DWI) Low B Axial	Maximum Length All Sequences	Pathology gross Tissue Measurements
**# of Cases Available for analysis**	49	48	49	33	42	42	25	52	52
**Average Length ± SD (cm)**	2.47 ± 1.25	2.65 ± 1.38	2.45 ± 1.12	2.51 ± 0.91	2.64 ± 1.32	2.62 ± 1.29	2.59 ± 1.37	2.96 ± 1.29	2.60 ± 1.57
**MRI compared to pathology** **CCC (95% CI)**	0.76 (0.62–0.85)	0.83 (0.72–0.90)	0.74 (0.61–0.84)	0.80 (0.62–0.89)	0.74 (0.58–0.85)	0.74 (0.57–0.84)	0.66 (0.39–0.82)	0.82 (0.71–0.89)	NA
**MRI compared to pathology** **Bland-Altman Bias (limits of agreement)**	−0.13 (−2.08, 1.83)	−0.05 (−1.76, 1.66)	−0.10 (−2.00, 1.80)	0.003 (−1.27, 1.28)	−0.07 (−2.18, 2.04)	−0.10 (−2.22, 2.03)	−0.33 (−2.29, 2.28)	0.34 (−1.25, 1.94)	NA
**MRI interobserver comparison** ICC (95% CI) *	0.82 (0.70–0.89)	0.83 (0.73–0.90)	0.80 (0.56–0.89)	0.74 (0.38–0.86)	0.83 (0.72–0.89)	0.83 (0.72–0.89)	0.91 (0.81–0.95)	0.76 (0.59–0.86)	NA
Maximum MRI exam diameter occurrence per sequence **	7	9	9	14	15	9	3	NA	NA

* *p* < 0.001 for all observations. ** if >1 series had the maximum diameter, each of these series is credited. W: Weighted; FS: Fat saturated; MRI: Magnetic resonance imaging; MRCP: Magnetic resonance cholangiopancreatography; SD: Standard deviation; CCC: Concordance correlation coefficient. NA: Not applicable; ICC: Intraclass correlation coefficient. CI: Confidence interval.

**Table 3:** MRI agreement with pathology grade.

	T2w Axial	T2w FS Axial	T2w Coronal	MRCP Coronal	T1w Arterial Axial	T1w Venous Axial	Diffusion Weighted Imaging (DWI) Low B Axial
MRI agreement with pathologic tumor grade Kappa (95%CI) *p* value	0.60 (0.37,0.83), *p* < 0.001	0.60 (0.37, 0.83), *p* < 0.001	0.60 (0.37, 0.83), *p* < 0.001	0.57 (0.30, 0.85), *p* < 0.001	0.50 (0.22,0.78), *p* = 0.001	0.45 (0.17, 0.73), *p* = 0.002	0.52 (0.22, 0.81), *p* = 0.003
MRI interobserver variability—1 or more WF/HRS Kappa (95%CI) *p* value	0.73 (0.52–0.93), *p* < 0.001	0.73 (0.52–0.93), *p* < 0.001	0.73 (0.52–0.93), *p* < 0.001	0.69 (0.45–0.94), *p* < 0.001	0.57 (0.30–0.83), *p* < 0.001	0.57 (0.30–0.83), *p* < 0.001	0.68 (0.39–0.96), *p* = 0.001

W: Weighted; FS: Fat saturated; MRI: Magnetic resonance imaging; MRCP: Magnetic resonance cholangiopancreatography; WF/HRS: worrisome features/high-risk stigmata; CI: Confidence interval.

## Data Availability

The data presented in this study are available on request from the corresponding author. The data are not publicly available due to [database complexity and local storage guidelines].
